# Evaluation of bacteriophage as an adjunct therapy for treatment of peri-prosthetic joint infection caused by *Staphylococcus aureus*

**DOI:** 10.1371/journal.pone.0226574

**Published:** 2019-12-26

**Authors:** Jodie L. Morris, Hayley L. Letson, Lisa Elliott, Andrea L. Grant, Matthew Wilkinson, Kaushik Hazratwala, Peter McEwen

**Affiliations:** 1 Orthopaedic Research Institute of Queensland, Townsville, Queensland, Australia; 2 College of Medicine and Dentistry, James Cook University, Queensland, Australia; 3 AusPhage Pty Ltd, Townsville, Queensland, Australia; Laurentian, CANADA

## Abstract

Phage therapy offers a potential alternate strategy for the treatment of peri-prosthetic joint infection (PJI), particularly where limited effective antibiotics are available. We undertook preclinical trials to investigate the therapeutic efficacy of a phage cocktail, alone and in combination with vancomycin, to reduce bacterial numbers within the infected joint using a clinically-relevant model of *Staphylococcus aureus*-induced PJI. Infected animals were randomised to 4 treatment groups, with treatment commencing 21-days post-surgery: bacteriophage alone, vancomycin alone, bacteriophage and vancomycin, and sham. At day 28 post-surgery, animals were euthanised for microbiological and immunological assessment of implanted joints. Treatment with phage alone or vancomycin alone, led to 5-fold and 6.2-fold reductions, respectively in bacterial load within peri-implant tissue compared to sham-treated animals. Compared to sham-treated animals, a 22.5-fold reduction in *S*. *aureus* burden was observed within joint tissue of animals that were administered phage in combination with vancomycin, corresponding with decreased swelling in the implanted knee. Microbiological data were supported by evidence of decreased inflammation within the joints of animals administered phage in combination with vancomycin, compared to sham-treated animals. Our findings provide further support for phage therapy as a tolerable and effective adjunct treatment for PJI.

## Introduction

Bacteriophage, or phages, are naturally occurring, obligate predators of bacteria that were discovered in the early 20^th^ century [1]. Phage safety in humans is well documented with phage therapy used in Eastern Europe for almost a century for the treatment of acute and chronic bacterial infections [2–4]. The continued and rapid global emergence of antibiotic resistance due to overuse and misuse prompted the recent World Health Organisation warning of this silent pandemic and its imminent dire implications without immediate changes to antimicrobial stewardship and identification of alternative treatment strategies [5]. Consideration of phages as an alternate therapeutic approach for difficult to treat bacterial infections, including osteomyelitis, has renewed over the past decade with an increase in the number of published case reports of successful clinical outcomes following phage therapy [6, 7] and a small number of clinical trials completed, or in progress, in the USA, United Kingdom and Australia (https://anzctr.org.au/, https://clinicaltrials.gov/, and https://globalclinicaltrialdata.com/) [3, 7]. Nonetheless, statistical evidence of phage therapy efficacy has been inconsistent with clinical trials completed to date, prompting calls for continued translational research efforts in this field so as to guide effective clinical practice in future [7].

Prosthetic joint infection (PJI) following total knee arthroplasty (TKA) remains the leading cause for revision surgery, with methicillin-susceptible *Staphylococcus aureus* (MSSA) the bacterium most frequently responsible [8–10]. *S*. *aureus* biofilm formation is a key component in the virulence armamentarium of this bacterium in the pathogenesis of PJI. Bio-inert orthopaedic materials such as titanium provide habitable substrates for biofilm formation, a growth state which serves to facilitate bacterial survival in hostile environments [11, 12]. Not only does the structure of the biofilm limit the penetration of antibiotics and immune mediators, but recalcitrance to treatment is also driven by altered metabolic phenotype of bacterial cells within the biofilm matrix [12–14]. Consequently, current surgical and antibiotic management strategies for PJI are not only costly and traumatic for the patient, but also associated with considerable morbidity and mortality with failure rates of 14.8% to 25% [8, 15–17].

In contrast to antibiotics which decrease in concentration below the surface of bacterial biofilms, phages are capable of penetrating biofilms and self-replicating [2]. To overcome potential limitations arising from the high phage specificity, phage cocktails have been used to broaden the spectrum of activity [2, 18, 19]. Recently, a French case series was published describing the successful treatment of PJI caused by *S*. *aureus* with phages in combination with antibiotics [18]. However, while the application of phages for the treatment of bone and joint infections appears promising, detailed preclinical and clinical studies to evaluate their *in vivo* efficacy are lacking. To our knowledge, there have been no preclinical studies using models that are clinically representative of TKA to investigate phage as an adjunct therapy for PJI caused by *S*. *aureus*.

Proof-of-principle *in vitro* studies demonstrated potential application of a lytic phage cocktail to reduce *S*. *aureus* numbers within biofilms growing on custom 3D-printed, miniaturized porous titanium implants, a material commonly used in the manufacture of orthopaedic implants [20]. Using the same titanium implants and additional biomaterials employed in modern TKA, a novel rat model of *S*. *aureus* biofilm-associated PJI was also recently characterised [21]. The aim of the current study was to compare the *in vivo* efficacy of single or combination therapy using a bacteriophage cocktail and vancomycin for reducing *S*. *aureus* burden within the implanted knee of the rat model of *S*. *aureus* biofilm-associated PJI. We hypothesised that *S*. *aureus* burden will be reduced in peri-implant tissue from animals receiving bacteriophage therapy, and that combination therapy with bacteriophage and vancomycin will further reduce bacterial load.

## Materials and methods

### Ethics statement

The care and use of all animals in this study was in strict accordance to the National Health and Medical Research Council Australian Code for the Care and Use of Animals for Scientific Purposes. All animal experimental procedures were approved by the James Cook University Institutional Animal Ethics Committee (A2326). Surgeries were performed under isoflurane anaesthesia and animal sacrifice was performed by pentobarbital (100 mg/kg) overdose at designated end-points.

### Microorganism and antimicrobial agents

A previously described MSSA clinical isolate, ORI16_C02N, recovered from a patient with delayed-onset PJI following TKA was used in the current study [20]. A single stock preparation was cultured overnight in tryptic soya broth (TSB), and stored in multiple, snap-frozen aliquots at -80°C. Aliquots from this master stock were used for all animal infection experiments. Sterile titanium implants were pre-seeded with *S*. *aureus* under *in vitro* conditions prior to implantation into rat femurs, as described previously [21]. The mean bacterial density on each implant at the time of surgery (day 0) was 1.2 x10^6^ CFU (range, 8.9 x 10^5^–1.9 x 10^6^ CFU).

Five lytic *S*. *aureus*-specific phages (StaPh_1, StaPh_3, StaPh_4, StaPh_11 and StaPh_16) of the family, *Myoviridae*, were used to prepare the StaPhage cocktail for the current study [20]. *In vitro* spot tests were performed to confirm bactericidal activity of each phage toward OR16_C02N [20]. Individual phage suspensions contained 1 x 10^9−12^ plaque-forming units (PFU)/mL and were stored at 4°C, with titres and sterility confirmed prior to each use. For preparation of StaPhage cocktail, individual phage preparations were adjusted 5 x 10^8^ PFU/mL in sterile 0.9% saline, then combined at equal volumes to achieve a cocktail containing 2.5 x 10^9^ PFU/mL. StaPhage cocktail was prepared immediately prior to administration to animals.

Vancomycin powder (Alphapharm, Sydney, Australia) was dissolved in sterile water and administered intraperitoneally at a dose of 50 mg/kg, as described below. The sensitivity of OR16_C02N to vancomycin was confirmed by the epsilometer (Etest) method (BioMérieux, Norwest, Australia) prior to the experiment (minimum inhibitory concentration, MIC 1.8 μg/mL).

### Animals

Conventional, twenty-week-old male Sprague-Dawley rats (n = 47, 360 to 480 g) were used. Animals were individually caged, fed a standard pellet diet and provided water *ad libitum*. After a 7-day acclimation period, knee implant surgery was performed on rats under isoflurane anaesthesia using surgical techniques and materials, as described previously [21]. Surgeries were performed on 10 to 12 animals per surgery day. Prior to each surgery day, animals were randomised to a treatment group, with equal numbers of animals per treatment group for each surgery date. The order of animals undergoing surgery on each surgery day was also randomised. Briefly, a UHXLPE implant was seated in a small mantle of gentamicin-laden bone cement (Heraeus Palacos® R+G, Zimmer Biomet, Sydney, Australia) within the proximal tibia. A titanium implant, pre-seeded with *S*. *aureus*, was press-fit into a defect created in the distal femur. Following implantation, the patella was repositioned, and the capsule and skin closed. Immediately after skin closure and prior to recovery from anaesthesia, animals received pre-emptive analgesic consisting of a 0.05 mg/kg subcutaneous injection of buprenorphine (Temgesic®) in a 1mL bolus of saline. Post-operative analgesic (buprenorphine, 0.01–0.05 mg/kg) was administered at 6 and 12 hrs post-surgery, with analgesic administered 12 hourly thereafter, according to pain scores of individual animals. Clinical signs including body weight, temperature and weight-bearing activity were monitored daily throughout the experimental period. Establishment of infection was confirmed by differential white blood cell at day 5 post-surgery (S1 Table) [21]. Animals were sacrificed at 28 days post-surgery (7 days after commencing treatment) with an overdose of pentobarbital (100 mg/kg) for gross pathology, haematology, microbiology, inflammatory, and histological evaluation. All animal experimental procedures were approved by the Institutional Animal Ethics Committee (A2326).

### Treatment

**Fig 1:**
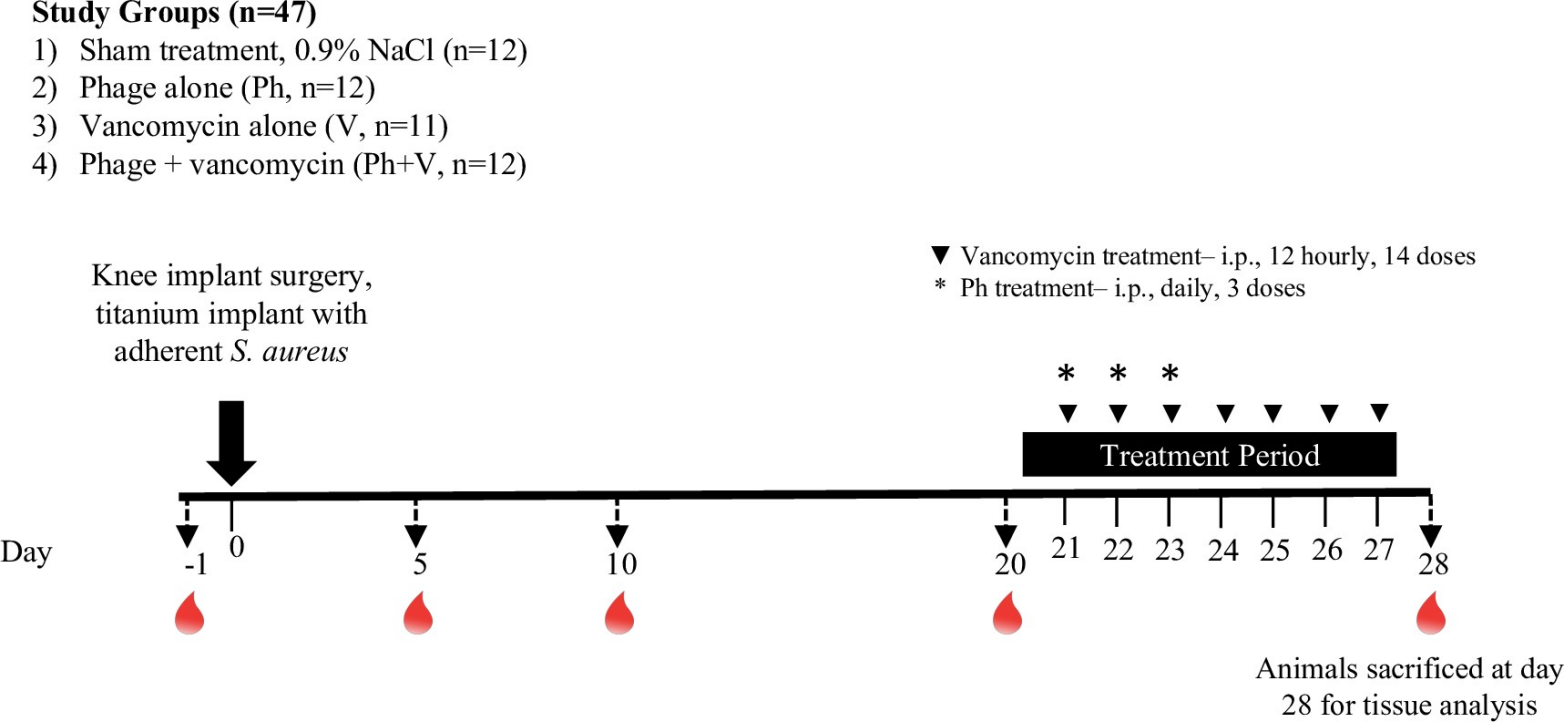
Schematic representation of the study protocol for evaluation of bacteriophage therapy in a rat model of *S*. *aureus* biofilm-associated PJI. Blood sampling via the lateral tail vein (indicated by droplet) was performed 1 day prior to surgery, and at day 5, 20 and 28 post-surgery. End-point analyses were performed at day 28 post-surgery (7 days after commencement of treatment).

At commencement of the 7-day acclimation period prior to surgery, animals were randomised to the following treatment groups: no treatment (Sham, n = 12), phage alone (Ph, n = 12), vancomycin alone (V, n = 11), phage plus vancomycin (Ph+V, n = 12) (Fig 1). Normal (0.9%) sterile saline was administered to sham-treated controls via the intraperitoneal (i.p.) route on day 21, 22 and 23 post-surgery. StaPhage cocktail (1.3 x 10^8^ PFU; multiplicity of infection (MOI), > 10^4^ PFU:1 CFU) was administered via the i.p. route on day 21, 22 and 23 post-surgery. Vancomycin (50 mg/kg, i.p.) was administered from day 21 to 27 post-surgery every 12 h [22–24]. Animals receiving combination therapy were administered phage and vancomycin in accordance with the single therapy regimens.

### Haematology and inflammatory assessments

Blood was collected under anaesthesia via the lateral tail vein during the experimental period and via terminal cardiac puncture at day 28 post-surgery (Fig 1). Complete blood cell examination (CBE) was carried out using an automated ACT Diff analyser (Beckman Coulter, Brea CA, USA). Blood samples were centrifuged, and plasma collected and stored at -80°C until analysis. C-reactive protein (CRP) was measured in plasma using a Rat C-reactive protein ELISA (BD Biosciences, North Ryde, Australia) and calprotectin was measured in joint tissue homogenates using a Rat Calprotectin ELISA (Cusabio, Houston, TX, USA), according to manufacturer protocols. Inflammatory chemokines and cytokines (MCP-1, GRO/KC, MIP-2, TNF-a, IL-1ß, IL-6, IL-12p70, IFN-g, IL-4, IL-10) were measured in plasma and joint tissue homogenates using Milliplex® Rat Cytokine/Chemokine Magnetic Bead Panel (RECYTMAG-65K, Lot #3070574 and #2927420, Abacus ALS, Meadowbrook, Queensland) in combination with the Magpix® analyser (Luminex Corporation, Austin, Texas, USA). Assays were carried out according to manufacturer’s instructions with samples measured in duplicate.

### Microbiological analyses

Bacterial loads were determined in blood, tissues and implants from animals at day 28 post-surgery, using methods described previously [21]. Briefly, liver, spleen, popliteal and inguinal (draining) lymph nodes of the operated (right leg) and non-operated (left leg) limb were dissected aseptically and weighed. The operated limb was also removed at the hip and soft tissue dissected from the femur and tibia. Bone cuts were made approximately 2.5 cm proximal to knee on femur and 2.5 cm distal on tibia. Titanium and UHXLPE implants were removed and sonicated as described previously Lytic bacteriophages replicate within a specific host bacterium causing lysis and death of the bacterial cell and release of phage progeny which perpetuate the cycle as long as live bacterial target cells are present., with UHXLPE implants removed from bone cement mantles prior to sonicating. After removal of implants, distal femur and proximal tibia pieces were weighed, pulverised and homogenised in sterile phosphate buffered saline (PBS, pH 7.2) [21]. In consideration of the potential for inadvertent haematogenous seeding of implants by respiratory or gut flora following surgical stress in the animals, tissue homogenates and implant sonicates were serially diluted in sterile PBS and cultured in triplicate on both selective (mannitol salt agar, MSA) and non-selective (tryptic soy agar, TSA) agar overnight to assess purity and to observe any phenotypic changes in colony morphology of the *S*. *aureus* isolates recovered from the infected joints. The limit of detection for viable counts was <5 CFU. Data is expressed as mean log_10_ CFU/total tissue or mean log_10_ CFU, as indicated.

### Antimicrobial sensitivity

Vancomycin and StaPhage sensitivity was determined for *S*. *aureus* (5–10 colonies) recovered from femur and titanium implants of treated and untreated animals at day 28 post-surgery. The susceptibility of the stock (pre-implantation) strain of ORI16-02N and isolates recovered from animals (n = 8 animals per treatment group) toward the individual phages and the StaPhage cocktail was tested using standard spot tests, as described previously [20]. Briefly colonies were recovered from TSA with a sterile cotton-tip swab, suspended in sterile TSB with 20% glycerol (v/v) and aliquots snap-frozen at -80°C. To assess antimicrobial sensitivity, single-use aliquots were thawed, diluted in sterile TSB (1:5) and grown to log-phase (3 h, 37°C, 100 rpm) prior to preparation of bacterial lawns on TSA. Bacterial sensitivity to phage was considered where confluent, semi-confluent, opaque lysis or individual plaques were observed in the bacterial lawn. A semi-quantitative scoring system (S1 Fig) was used to enable comparison of phage sensitivity between isolates where: 0 = no plaques, 1 = < 10 individual plaques, 2 = 11–100 individual plaques, 3 = partial clearing, >100 plaques, 4 = clearing with several distinct bacterial colonies within the clearance zone, 5 = complete clearing. Vancomycin susceptibility of *S*. *aureus* recovered from femur and titanium implants was compared to the pre-implantation *S*. *aureus* strain using the Etest method, and according to manufacturer protocols.

### Statistics

Statistical analyses were performed using GraphPad Prism for Mac software (version 7). Data normality was assessed using Shapiro-Wilks test, with Levene’s test used to determine equality of variances. Non-normally distributed data was compared using a Mann-Whitney U test or Kruskal-Wallis test with Dunn’s post-hoc analysis. Changes in haematology parameters between groups were compared using two-way repeated measures ANOVA, with Holm-Sidak post-hoc analysis. Within group differences were analysed with repeated measures ANOVA with Dunnet’s post-hoc analysis. MILLIPLEX Analyst 5.1 software (Luminex Corporation, Austin, Texas, USA) was used to determine cytokine and chemokine concentrations with a 5-parametric logistic weighted curve fit. Results are expressed as mean ± standard deviation (SD) unless otherwise stated, with significance set at P < 0.05.

## Results

### Clinical outcomes

All animals survived surgery and the postoperative period with no signs of systemic illness. Administration of analgesic was ceased by day 5 post-surgery for all animals based on improved clinical scores. Body temperature increased significantly between baseline and day 5 post-surgery for all animals, though levels remained within normal ranges throughout the experimental period with no discernible effects of treatment on body temperature (S1 Table) [21]. Animals were able to bear partial weight within 48 h of surgery. The median time to full weight bear was 18 days post-surgery, with 12 of 47 animals not returning to full weight-bearing within the 28-day experimental period. No significant difference was observed in the time for sham-treated animals (median 21 d, range 10–28 d), or animals treated with phage alone (median 18 d, range 13–28 d), vancomycin alone (median 17 d, range 11–28 d) or combined phage and vancomycin (median 18 d, range 11–28 d) to return to full weight bearing following surgery.

Minor weight loss was observed for all animals in the first week following surgery, however body weight increased thereafter (S1 Table). Compared to sham-treated controls, body weight was significantly lower in animals treated with phage alone, vancomycin alone and phage plus vancomycin at day 28 post-surgery, despite comparable pre-treatment weights at day 20 post-surgery (S1 Table).

### Haematology and systemic inflammation

There were no statistically significant differences in haematology parameters between treatment groups at baseline, or throughout the experimental period (S1 Table). While significant changes were observed in erythrocyte, leucocyte and platelet parameters for all animals following surgery, numbers tended to return to pre-surgical levels by the end of the experimental period, consistent with previous findings in this infection model [21]. Establishment of infection was confirmed for all animals by a significant increase in the percentage of neutrophils at day 5 post-surgery as previously described [21]. Similarly, plasma CRP concentrations were significantly increased at day 5 post-surgery for all animals, though concentrations returned to baseline levels by day 20 post-surgery and were comparable between treatment groups at day 28 post-surgery (S1 Table). Plasma lactate concentrations (indirect marker of tissue hypoxia) were significantly increased at day 28 post-surgery compared to pre-surgical levels for all treatment groups however, no significant between-group differences observed (S1 Table).

**Fig 2:**
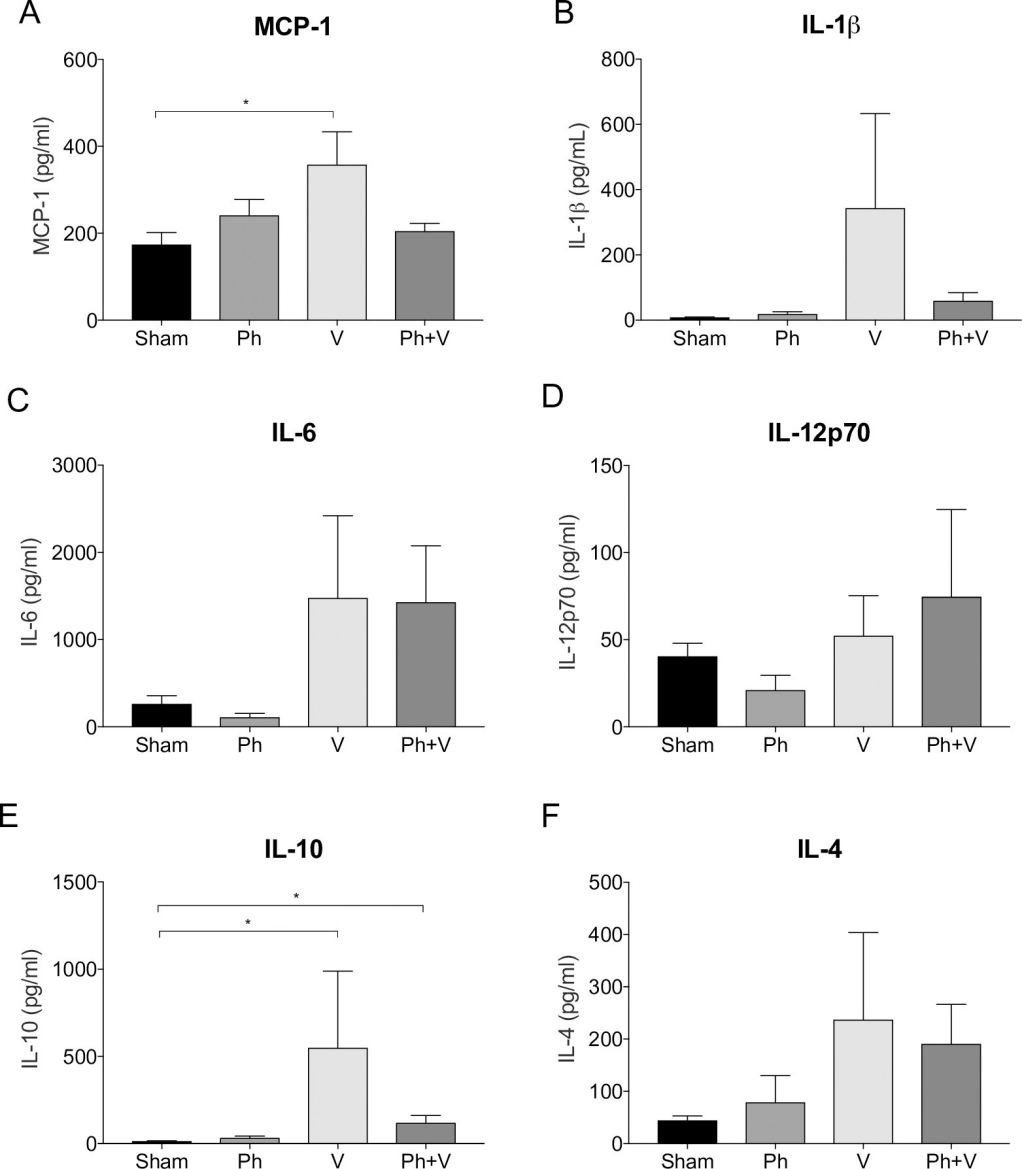
Systemic inflammatory mediators. Concentrations of MCP-1, IL-1ß, IL-12p70, IL-6, IL-10 and IL-4 were measured in plasma of sham-treated (Sham, n = 10) and treated (Ph, phage alone, n = 8; V, vancomycin alone, n = 8; Ph+V, phage plus vancomycin, n = 9) at day 28 post-surgery (7 days after commencement of treatment). Data shows mean ± SD. *P < 0.05.

Plasma inflammatory chemokine and cytokine levels were comparable between treatment groups at day 28 post-surgery (7 days after commencing treatment; Fig 2). TNF-a and IFN-g levels remained below the assay limit of detection for all animals (<2.4 pg/ml and <14.6 pg/ml, respectively). No significant differences were observed between sham-treated and phage-treated animals for any of the inflammatory mediators measured at day 28 post-surgery. Compared to sham-treated animals, plasma IL-10 levels were significantly higher in animals treated with vancomycin alone and combined phage and vancomycin at day 28 post-surgery (p = 0.04 and p = 0.001, respectively; Fig 2E). Similarly, MCP-1 levels were significantly elevated in plasma from vancomycin-treated animals compared to sham-treated controls (p = 0.018; Fig 2A). No significant differences were observed in IL-6, IL-1ß and IL-4 concentrations in plasma of vancomycin-treated and phage plus vancomycin-treated animals at day 28 post-surgery compared to sham-treated controls (Fig 2B, 2C and 2F).

### Joint swelling and pathology

**Fig 3:**
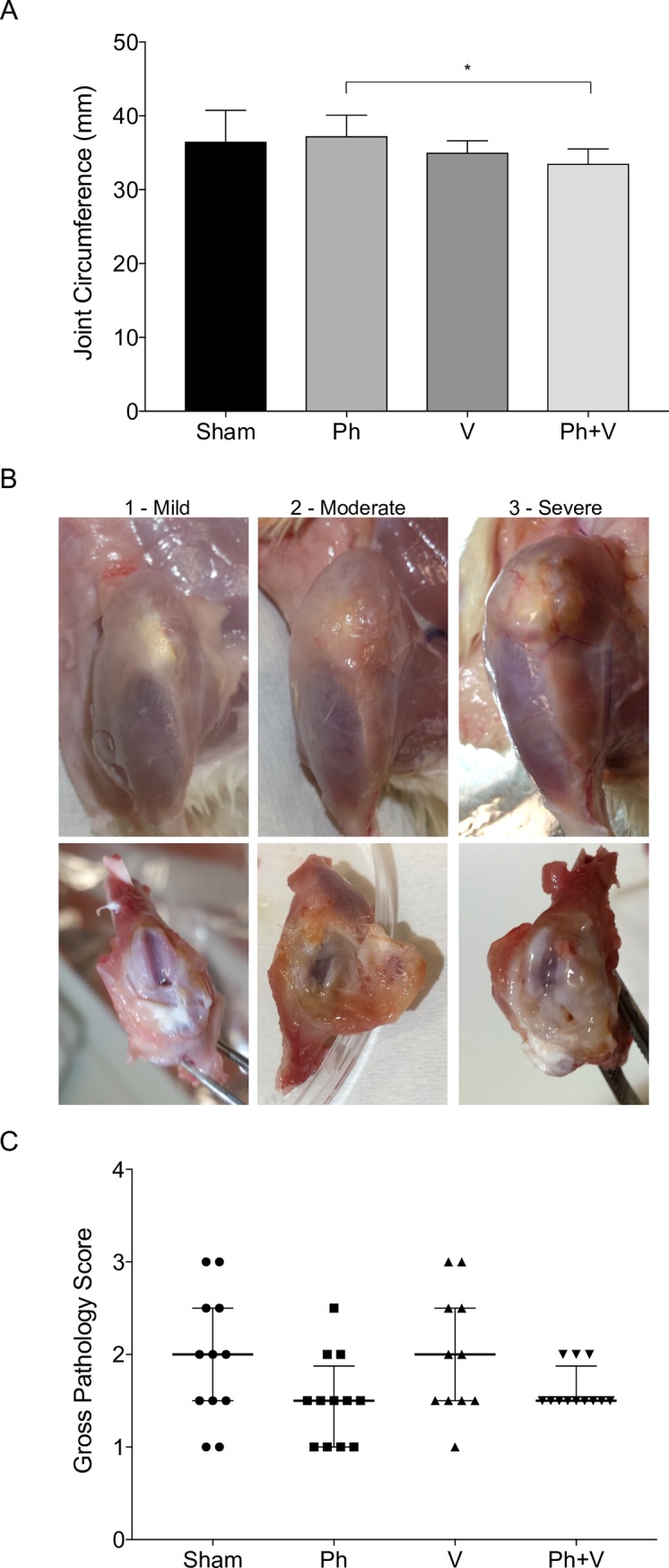
Joint swelling and pathology. A) Joint circumference of the operated (implant) limb of infected and sham-treated (Sham, n = 12), and those treated with phage alone (Ph, n = 12), vancomycin alone (V, n = 11) and phage plus vancomycin (Ph+V, n = 12) at day 28 post-surgery. Data shows mean ± SD. *P < 0.05 B. Representative images of mild (1), moderate (2) and severe (3) joint pathology changes in the operated hind limb of infected animals at day 28 post-surgery. Based on this scoring system, C) gross joint pathology scores were compared for sham-treated (Sham), and those treated with phage alone (Ph), vancomycin alone (V) and phage plus vancomycin (Ph+V) at day 28 post-surgery (7 days after commencement of treatment). Data shows median ± interquartile range.

Surgical incisions healed without complication in all animals. Joint circumferences of implanted knees were comparable between sham-treated, phage-treated and vancomycin-treated animals (Fig 3A). Compared to treatment with phage alone, the joint circumference of implanted knees was significantly less for animals receiving combination therapy (p = 0.016; Fig 3A). Upon dissection, macroscopic examination of the operated knees of animals at day 28 post-surgery revealed mild to severe soft tissue and articular cartilage damage, often in combination with increased amount and viscosity of synovial fluid (Fig 3B). A numerical scoring system was used for semi-quantitative evaluation of gross pathology within operated joints of animals at day 28 post-surgery, where 1 = mild soft tissue and articular cartilage damage and the absence of synovial effusion; 2 = moderate soft tissue changes, synovial thickening, occasional focal damage to articular cartilage with synovial effusion; and 3 = severe and extensive soft tissue damage, synovial thickening, articular cartilage damage and synovial effusion with Baker’s cyst (Fig 3B). No significant differences were observed in joint pathology between treatment groups, though there was a trend for reduced tissue damage within joints from animals that received combination therapy (p = 0.13; Fig 3C).

### Joint bacterial loads

**Fig 4:**
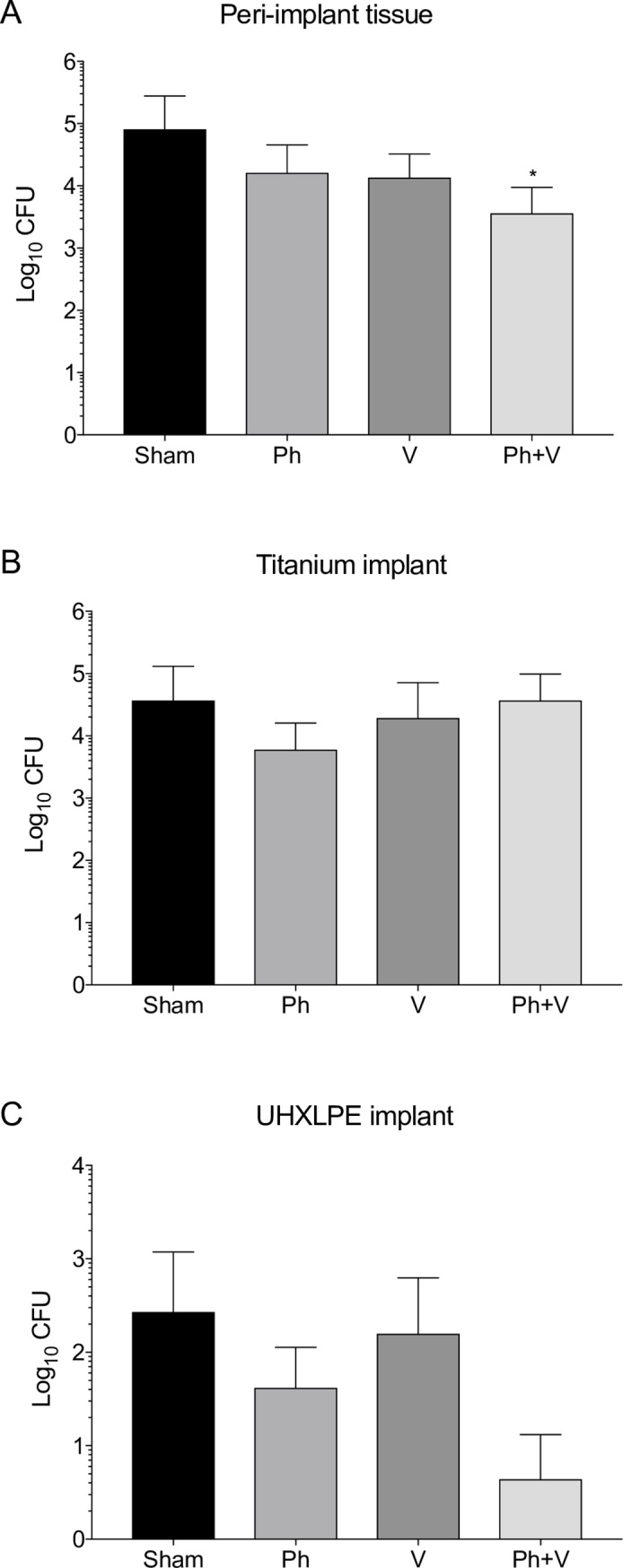
The effect of single and combination antimicrobial therapy on *S*. *aureus* numbers in implanted knees. Phage alone (Ph, n = 12), vancomycin (V, n = 11) or phage plus vancomycin (Ph+V, n = 12) was administered intraperitoneally to rats from day 21 post-surgery. Sham-treated (Sham) animals were administered an equivalent volume of sterile 0.9% saline. Peri-implant joint and bone tissue and implants were harvested from animals 7 days after commencement of antimicrobial therapy (28 days post-surgery) for enumeration of bacteria. The mean CFU count for the A) peri-implant tissue homogenates for the entire joint (a sum of the femur, tibia, patella and surrounding capsular tissue) following removal of implants, B) the titanium implants and C) the UHXLPE implants was determined for each treatment group. Data shows log_10_ CFU/tissue and log_10_ CFU/implant ± SD. *P < 0.05 compared to sham-treated animals.

Seven days after commencement of antimicrobial therapy, peri-implant joint tissue (femur, tibia, patella and associated tendons, joint capsule tissues) and implants (titanium, UHXLPE) were harvested for enumeration of bacteria (Fig 4, Table 1). Peri-implant tissue weights were comparable between sham-treated (3.0 ± 0.5 g), and phage alone (3.0 ± 0.4 g), vancomycin alone (2.9 ± 0.3 g), and phage plus vancomycin (2.8 ± 0.4 g) treated animals (p = 0.632). Sham-treated animals had 8.1 x 10^4^ ± 2 x 10^5^ CFU (range, 8 x 10^1^–7 x 10^5^ CFU) in peri-implant joint tissue, and 3.7 x 10^4^ ± 9.4 x 10^4^ CFU (range, 1.5 x 10^2^–3.3 x 10^5^ CFU) recovered from titanium implants (12 of 12 animals). Treatment with phage alone resulted in 1.6 x 10^4^ ± 2.9 x 10^4^ CFU (p = 0.100; 5-fold reduction; range, 0–8 x 10^4^ CFU) in peri-implant tissue and 6 x 10^3^ ± 1 x 10^4^ CFU (p = 0.947; 6.2-fold reduction; range, 1.2 x 10^2^–3.7 x 10^4^ CFU) from titanium implants. Animals treated with vancomycin alone had 1.3 x 10^4^ ± 1.9 x 10^4^ CFU from peri-implant tissue (p = 0.235; 6.2-fold reduction; range, 0–5.5 x 10^4^ CFU) and 1.9 x 10^4^ ± 5.2 x 10^4^ CFU from titanium implants (p = 0.921; 1.9-fold reduction; range, 2.3 x 10^2^–1.8 x 10^5^ CFU). Combination therapy with phage and vancomycin significantly reduced *S*. *aureus* numbers to 3.6 x 10^3^ ± 5.8 x 10^3^ CFU (p = 0.014; 22.5-fold reduction; range, 0–1.6 x 10^4^ CFU) in peri-implant tissue. However, mean bacterial load on titanium implants was comparable to those in sham-treated animals (p = 0.852; 3.7 x 10^4^ ± 6.1 x 10^4^ CFU; range, 4.1 x 10^1^–1.6 x 10^5^ CFU). *S*. *aureus* was recovered from sonicated UHXLPE implants in sham-treated (4 of 12), phage alone (4 of 12), vancomycin alone (5 of 11), and phage plus vancomycin combination therapy (3 of 12). No significant differences were observed between mean bacterial numbers recovered from UHXLPE implants between treatment groups though numbers tended to be lower from animals treated with phage plus vancomycin, compared to sham-treated control animals (p = 0.365, Fig 4C; Sham, 2.7 x 10^2^ ± 9.1 x 10^2^ CFU *v* Ph+V, 4.4 x 10^0^ ± 8.8 x 10^0^ CFU).

**Table 1:** Proportions of *S*. *aureus* positive cultures within the joint by specimen type and treatment group.

Treatment Group	No. of positive cultures/Total number of samples		
	Femur	Tibia	Patella^
Sham	12/12 (100)	10/12 (83)	9/12 (75)
Ph	9/12 (75)	7/12 (58)	7/12 (58)
V	10/11 (91)	9/11 (82)	4/11 (36)
Ph+V	10/12 (83)	5/12 (42)	2/12* (17)

Number and percentage of joint tissue samples from which *S*. *aureus* was recovered at day 28 post-surgery in sham-treated (Sham), and those treated with phage alone (Ph), vancomycin alone (V) and phage plus vancomycin (Ph+V).

^includes surrounding tendons and capsular tissue. Statistical analyses were conducted using the Chi-square test.

*P < 0.05 compared to sham-treated.

*S*. *aureus* was recovered from the femur (12 of 12), tibia (10 of 12), patella and associated tendons and capsular tissue (9 of 12) of sham-treated animals at day 28 post-surgery (Table 1). In contrast, compared with sham-treatment, fewer animals were positive for *S*. *aureus* in tibia (ns, p = 0.098; 1.8-fold decrease), patella and joint soft tissue (p = 0.025; 4-fold decrease) following combination-therapy (Table 1).

### Effect of antimicrobial therapy on local inflammatory responses

**Fig 5:**
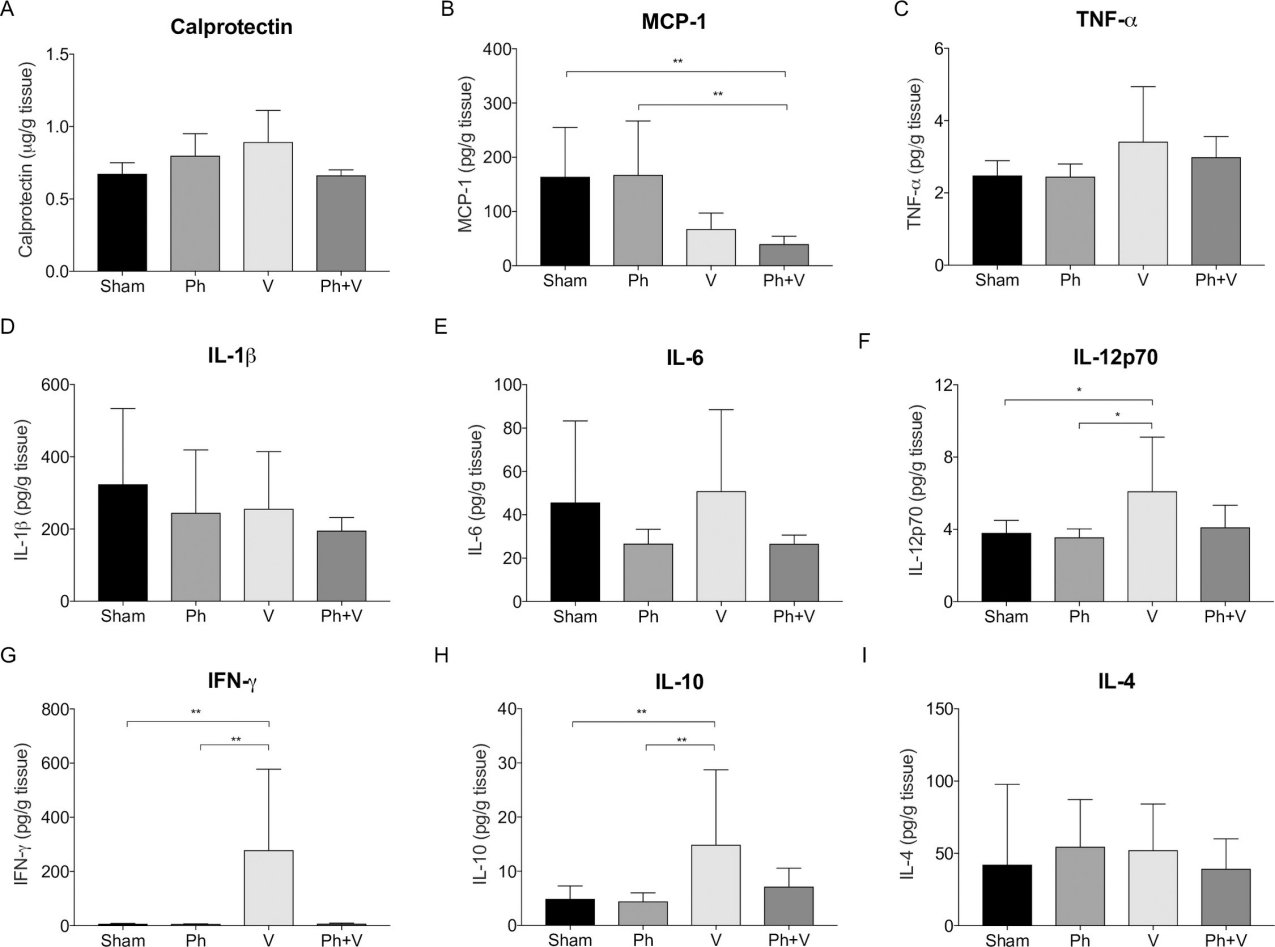
Inflammatory mediators in joint tissue. Concentrations of calprotectin, MCP-1, TNF-a, IL-1ß, IL-12p70, IFN-g, IL-6, IL-10 and IL-4 were measured in joint tissue of implanted knees of sham-treated (Sham, n = 10) and animals treated with phage alone (Ph, n = 10), vancomycin (V, n = 10) or phage plus vancomycin (Ph+V, n = 10) at day 28 post-surgery (7 days after commencement of treatment). Data shows mean ± SD. *P < 0.05.

Joint tissue concentration of calprotectin, an indirect marker of neutrophil infiltration, was comparable between sham-treated and single and combination therapy groups at day 28 post-surgery (Fig 5A). No significant differences were observed between sham-treated and phage alone treated animals for any of the inflammatory markers assayed. Compared to sham-treated animals and animals treated with phage alone, MCP-1 was significantly lower in joint tissue of animals treated with phage plus vancomycin at sacrifice (p = 0.0001 and p = 0.0002 respectively; Fig 5B). While differences were not statistically significant, there was a trend for decreased concentrations of MCP-1 in joint tissue from vancomycin alone treated animals (p = 0.06). Concentrations of TNF-a, IL-1ß, IL-6 and IL-4 were comparable between sham-treated, phage alone, vancomycin alone and phage plus vancomycin treatment groups (Fig 5C, 5D, 5E and 5I). Compared to sham-treated and animals treated with phage alone, significantly increased IL-12p70 (p = 0.033 and p = 0.003, respectively), IFN-g (p = 0.007 and p = 0.008, respectively) and IL-10 (p = 0.004 and p = 0.002) levels were measured in joint tissue from animals treated with vancomycin alone (Fig 5F, 5G and 5H).

### Development of antimicrobial resistance

Phage sensitivity was assessed to determine whether isolates recovered from the femur and excised titanium implants of animals (n = 8 per treatment group) at day 28 post-surgery were as susceptible as the original pre-inoculating *S*. *aureus* strain (S2 Table). StaPhage cocktail sensitivity of all *S*. *aureus* strains recovered from bone and titanium implants was comparable to that of the original, pre-implantation strain. However, screening of individual phage preparations identified phage-resistant colonies for isolates recovered from the femur of animals treated with phage alone, compared to the original *S*. *aureus* strain for StaPh_4, StaPh_11, and StaPh_16 (S2 Table). StaPh_1- and StaPh_3-resistant colonies were also observed for isolates recovered from bone and implants of sham-treated animals and animals that had been treated with vancomycin alone or phage plus vancomycin, respectively (S2 Table). No significant differences were observed in the sensitivity of *S*. *aureus* isolates from sham-treated and phage alone-treated animals toward StaPhage cocktail (p = 0.608), StaPh_1 (p > 0.999), StaPh_3 (p = 0.315), StaPh_4 (p = 0.132), StaPh_11 (p > 0.999), StaPh_16 (p > 0.999) (S2 Table). Within each treatment group, there was also no significant difference in phage sensitivity between strains recovered from bone and those from the titanium implant (S2 Table).

**Fig 6:**
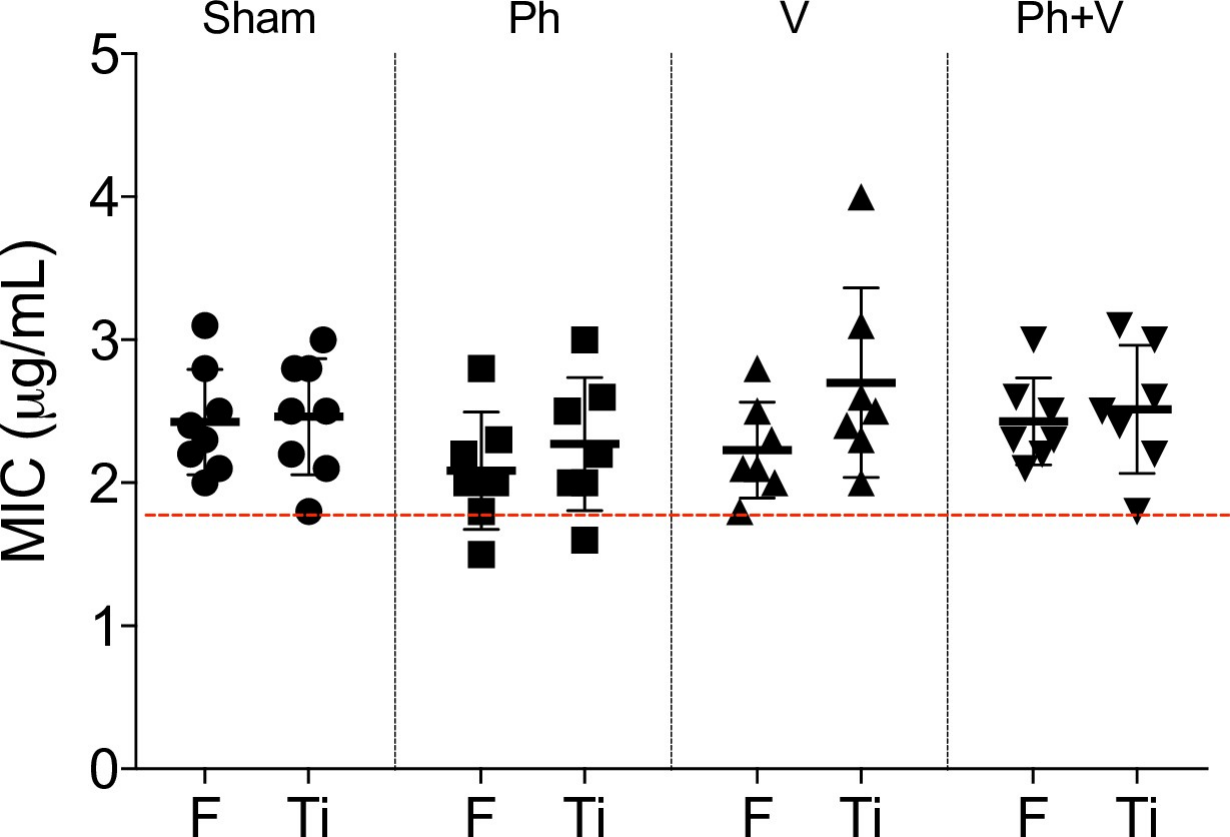
*In vitro* vancomycin susceptibility of *S*. *aureus* isolates recovered from the peri-implant tissue. *In vitro* vancomycin susceptibility of *S*. *aureus* isolates recovered from the femur (F) and the titanium implants (Ti) of sham-treated animals (Sham, n = 8) and animals treated with phage alone (Ph, n = 7), vancomycin alone (V, n = 7) or phage plus vancomycin (Ph+V, n = 7) at day 28 post-surgery was compared to the inoculating strain of *S*. *aureus* (dotted red line, n = 5 individual tests, mean MIC = 1.8 μg/ml). Data shows median ± interquartile range.

Vancomycin susceptibility of bacterial isolates recovered from the peri-implant tissue of the femur and the titanium implants of sham-treated animals (n = 8) and animals treated with phage alone (n = 7), vancomycin alone (n = 7) or phage plus vancomycin (n = 7) at day 28 post-surgery was compared to the inoculating strain of *S*. *aureus* (Fig 6). The MIC for ORI-16C02N (n = 5 independent tests) prior to implantation in the rat model of PJI was 1.8 μg/ml. The MIC for the majority of *S*. *aureus* isolates recovered from peri-implant tissue of sham-treated (p = 0.008), and animals treated with vancomycin alone (p = 0.02) and phage plus vancomycin (p = 0.03) were significantly higher than pre-infection levels. Similarly, significantly increased MICs were observed for *S*. *aureus* isolates recovered from titanium implants of sham-treated (p = 0.02), and animals treated with vancomycin alone (p = 0.02) and phage plus vancomycin (p = 0.03) compared to pre-infection levels. However, no significant differences were observed between vancomycin susceptibility of isolates from treated and untreated animals. Furthermore, *in vitro* vancomycin sensitivities were comparable between isolates recovered from peri-implant tissue and the surface of titanium implants within each treatment group (Fig 6).

## Discussion

As a looming post-antibiotic era threatens to undo a century of infectious diseases advancements, increasing consideration is once again being given to phages as an alternative approach for treating bacterial infections [3, 5]. Lytic bacteriophages replicate within a specific host bacterium causing lysis and death of the bacterial cell and release of phage progeny which perpetuate the cycle as long as live bacterial target cells are present. Due to the absence of cytotoxic effects toward mammalian cells, their high specificity, and their ability to infect bacterial cells within biofilms, phages could potentially be used as an adjunct to antibiotics for treatment of biofilm-associated PJI, although to date, preclinical studies evaluating their *in vivo* efficacy have been lacking [1–3, 18, 25]. Using a clinically-relevant rat model of S. *aureus* biofilm-associated PJI following TKA, we show several key findings in the present study. Firstly, the combination of phages with vancomycin exerted a significantly increased therapeutic benefit compared to single therapy. Secondly, phage therapy alone tended to reduce bacterial burden within joint tissue and on the titanium implant of the infected knee within one week of commencing treatment, though this did not reach statistical significance. Thirdly, no adverse local or systemic inflammatory effects were observed following administration of several doses of relatively high numbers of lytic phages. Finally, *S*. *aureus* isolates recovered from the infected knee of animals that received phage therapy remained susceptible to the five-phage cocktail.

The model used in the present study is clinically representative of delayed-onset PJI, where implant-associated biofilms and progressive inflammatory responses and tissue remodelling occur in peri-implant tissue over a period of weeks following surgery [21]. Though demanding, it is an appropriate translational model for evaluating *in vivo* antimicrobial efficacy against biofilm-associated PJI. Our data support synergism between phages and vancomycin, leading to significantly reduced bacterial burden within joint tissue within 7 days of commencing treatment. While not statistically significant, the trend observed for decreased bacterial numbers on titanium implants from animals exposed to phage alone may hold clinical significance. Immune cell infiltration was not directly measured in the present study however, levels of IFN-g, IL-12p70 and IL-10 in joint tissue of animals treated with vancomycin alone were significantly higher than in sham- and phage alone-treated animals, suggesting that bacterial reduction observed in this group was associated with increased activation of cell-mediated immune responses. In contrast, the similarities between calprotectin (indirect marker of neutrophil infiltration) and inflammatory mediator concentrations within peri-implant tissue from sham- and phage alone-treated animals suggests the reduction in bacterial load is directly attributed to bactericidal activity of phages, rather than immune activation *per se*.

Vancomycin pharmacokinetics in rodents have previously been described [26], and the dose used in the present study is sufficient to significantly reduce *S*. *aureus* numbers in experimental models of chronic osteomyelitis [22–24]. There is currently no standard regimen for phage administration for treatment of PJI. Previous experimental studies evaluating phage therapy for osteomyelitis have used doses of 10^7^−10^9^ PFU administered daily for 3 to 8 days [19, 27, 28]. We therefore chose to administer over 3 consecutive days, using a phage cocktail containing a high dose of phage (>10^8^ PFU). However, the effectiveness of phage therapy is dependent on the phage reaching the site of infection. Difficulties with eradication of chronic PJI arise due to poor vascular perfusion of ischaemic bone, the accumulation of peri-implant necrotic and fibrotic tissue and formation of sequestra which impede penetration of antimicrobials to the implant-associated biofilm [21, 29, 30]. It is possible that an extended therapeutic regimen may have led to further reduction in bacterial burden. The mechanisms underlying phage-antibiotic synergy and how these interactions evolve during the course of therapy and influence clinical outcome remains an area of active investigation [3, 31].

*In vivo* persistence of phages at day 28 post-surgery (4 days after final administration of phage cocktail) was confirmed in representative animals treated with phage and vancomycin (Ph+V, n = 5) by standard spot tests with 10-fold dilutions of femur homogenates onto confluent growth of the propagating strains of *S*. *aureus* (AP029 and AP030) for the lytic phages used in the study [32]. Further support for phage accumulation and replication within the infected knee following i.p. administration is provided by the high phage doses administered, the reduction in joint tissue bacterial loads 7 days after administration of phages, and the absence of significant local inflammatory responses compared to administration of vancomycin alone. Phage replication and *in vivo* persistence following systemic administration with doses comparable to those used in the current study has also been well-documented [2, 33]. However, we acknowledge that there may be differences in the proportion of each of the individual phage preparations reaching the site of infection, and the contribution of each in reducing *S*. *aureus* numbers.

Scheduling is an important consideration for phage therapy, particularly in relation to the impact on innate and adaptive immune responses [34, 35]. Whilst phages cannot infect nor replicate within eukaryotic cells, they do interact and influence eukaryotic cell activity, including immune cells. Opsonisation of bacteria with phages for example, has been shown to increase bacterial uptake by phagocytes [34, 36]. Strategies to improve the *in vivo* persistence of phages following systemic delivery are actively being investigated and include encapsulation with polymeric microparticles and liposomes [4, 36]. Administration of phages by alternate routes, including oral, aerosol and perioperative delivery has also shown to be effective for treatment of chronic bacterial infections [7]. Further studies to elucidate the optimal approach for phage administration and the mechanisms underlying the success or failure of phage therapy for biofilm-associated PJI are warranted.

The potential emergence of phage-resistant variants following phage therapy is recognised, with bacterial strains evolving strategies to block adsorption, prevent injection of phage genetic material and interrupt other systems used by phages to replicate [37, 38]. However, the clinical significance of phage-resistant variants remains unclear. A number of studies have shown that phage resistance may diminish fitness or virulence of these bacterial variants and therefore facilitate clearance by the immune system [38]. In addition to the role of selective pressure in emergence of bacterial resistance, phage-resistant bacterial variants can also occur spontaneously [38]. This was observed in the present study for *S*. *aureus* isolates recovered from bone and implants of sham- and vancomycin-treated animals. Notably, phage therapy was not associated with the emergence of resistant variants toward the five-phage cocktail. Indeed, the use of therapeutic phage cocktails is recommended for this very reason: variants resistant to one specific phage type, may remain susceptible to the others comprising the cocktail [38]. Similarly, vancomycin resistance was not observed following antibiotic administration in the present study, with MICs remaining below the MIC breakpoint for intermediate resistance [39]. Nevertheless, the mean vancomycin MIC for *S*. *aureus* strains recovered from animals in all treatment groups was significantly higher than the parent strain, suggesting that *in vivo* growth contributes to the emergence of genetic or phenotypic changes that influence vancomycin sensitivity. Selection for heteroresistance of MSSA to vancomycin (hVISA) and vancomycin MIC creep has been described for osteoarticular infections, though the clinical significance of these variants is not well understood [40, 41]. Further work to address the scientific and therapeutic complexities of phage therapy for the treatment of biofilm-associated PJI including dosing, route of administration and scheduling to improve delivery and efficacy at the site of infection is warranted.

In summary, our data support the concept of phage therapy as a safe and effective adjunct to antibiotics for treatment of *S*. *aureus* biofilm-associated PJI following TKA. Two-stage revision is currently the gold standard for management of delayed-onset or chronic PJI, though success rates remain suboptimal [8, 15–17]. One-stage revision is a preferred, more cost-effective approach, requiring a single surgery [42]. If phages could be used in combination with antibiotics, antimicrobial peptides and/or biofilm-disrupting enzymes to improve eradication of bacterial biofilms, one-stage exchanges and implant retention may be feasible. These findings lay the foundation for continued translational studies to optimise phage therapy as a viable adjunct for the treatment of PJI in a post-antibiotic era.

## Supporting information

S1 Fig Scoring of *in vitro* sensitivity of *S. aureus* toward StaPhage preparations. Representative images of the semi-quantitative scoring system used to assess the *in vitro S*. *aureus* susceptibility toward StaPhage preparations. 0 = no plaques, 1 = < 10 individual plaques, 2 = 11–100 individual plaques, 3 = partial clearing, >100 plaques, 4 = clearing with several distinct bacterial colonies within the clearance zone, 5 = complete clearing.
(TIF)

S1 Table Clinical and haematology parameters. Body weight, body temperature, haematology and systemic inflammation were assessed at baseline, prior to (day 5 and 20 post-surgery) and following treatment (day 28 post-surgery) for sham-treated (Sham) animals, and those treated with phage alone (Ph), vancomycin alone (V) and phage plus vancomycin (Ph+V).
(PDF)

S2 Table Phage sensitivity patterns of *S. aureus* isolates. Sensitivity screening of individual phage preparations and the StaPhage cocktail was performed for *S*. *aureus* isolates recovered from femur and titanium implants of sham-treated animals and animals treated with phage alone (Ph), vancomycin alone (V) or combination therapy (Ph+V) at day 28 post-surgery (n = 8 animals per group), and compared to that of the original, pre-implantation *S*. *aureus* strain.
(PDF)
