# Supplementary figures and images for: Evaluation of bacteriophage as an adjunct therapy for treatment of peri-prosthetic joint infection caused by Staphylococcus aureus
Source: PLoS One. 2019 Dec 26;14(12):e0226574. doi: 10.1371/journal.pone.0226574 (PMC6932802; doi:10.1371/journal.pone.0226574)

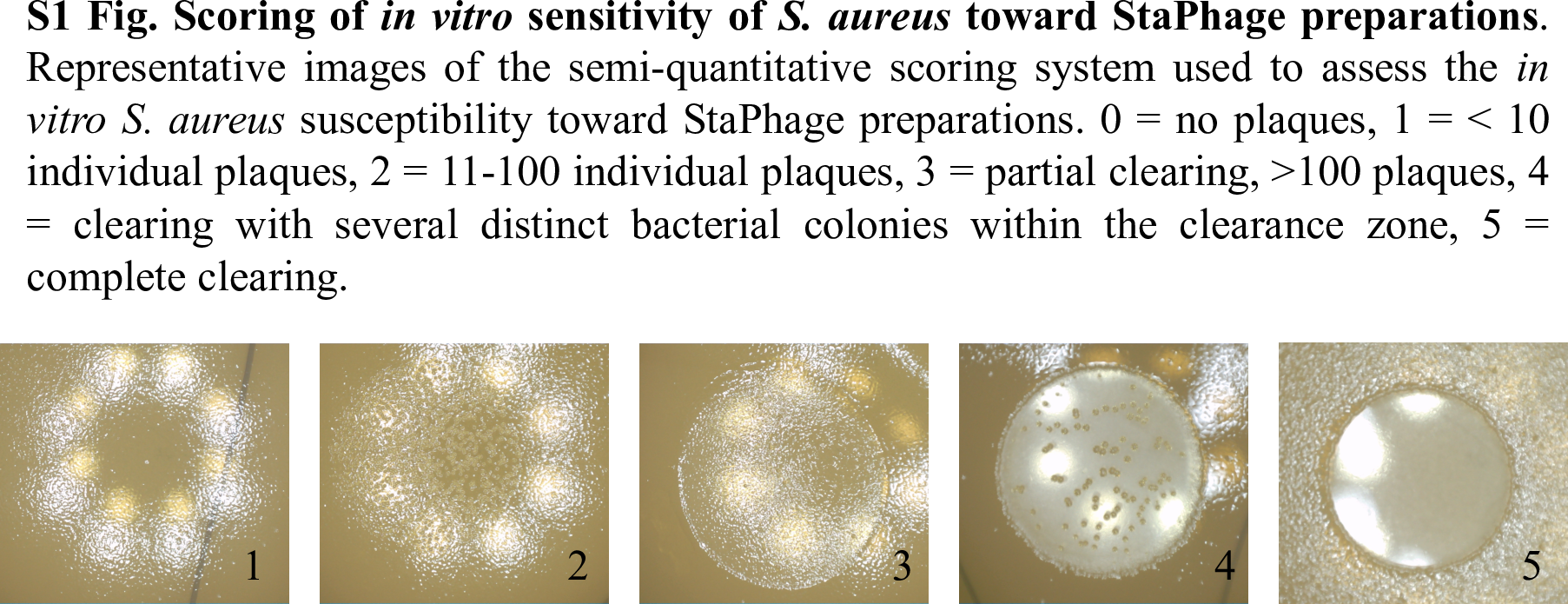

Supplement: S1 Fig — Representative images of the semi-quantitative scoring system used to assess the in vitro S. aureus susceptibility toward StaPhage preparations. 0 = no plaques, 1 = < 10 individual plaques, 2 = 11–100 individual plaques, 3 = partial clearing, >100 plaques, 4 = clearing with several distinct bacterial colonies within the clearance zone, 5 = complete clearing. (TIF) [file pone.0226574.s001.tif]
