# Supplementary material for: Evaluation of bacteriophage as an adjunct therapy for treatment of peri-prosthetic joint infection caused by Staphylococcus aureus
Source: PLoS One. 2019 Dec 26;14(12):e0226574. doi: 10.1371/journal.pone.0226574 (PMC6932802; doi:10.1371/journal.pone.0226574)
Supplement: S1 Table — Body weight, body temperature, haematology and systemic inflammation were assessed at baseline, prior to (day 5 and 20 post-surgery) and following treatment (day 28 post-surgery) for sham-treated (Sham) animals, and those treated with phage alone (Ph), vancomycin alone (V) and phage plus vancomycin (Ph+V). (PDF) [file pone.0226574.s002.pdf]

S1 Table. Clinical and haematology parameters. Body weight, body temperature, haematology and systemic inflammation were assessed at baseline, prior to (day 5 and 20 post-surgery) and following treatment (day 28 post-surgery) for sham-treated (Sham) animals, and those treated with phage alone (Ph), vancomycin alone (V) and phage plus vancomycin (Ph+V).

**Parameter**	**Treatment Group**	**Baseline**	**Pre-Treatment**		**Post-Treatment**	**P value**
		**day 0**	**day 5**	**day 20**	**day 28**	
Body weight, % change from baseline	Sham	-	-10.5 ± 2.3	-3.0 ± 3.5	1.1 ± 3.6	<0.001
	Ph	-	-11.4 ± 1.4	-5.5 ± 3.9	-3.5 ± 3.4*	
	V	-	-12.3 ± 2.0	-7.6 ± 6.4	-6.8 ± 2.5*	
	Ph+V	-	-12.8 ± 1.1	-2.3 ± 7.5	-5.9 ± 3.5*	
Body temperature, ^{o}C	Sham	35.8 ± 0.2	36.2 ± 0.3$^{\#}$	36.0 ± 0.3	35.8 ± 0.1	0.047
	Ph	35.8 ± 0.3	36.1 ± 0.2*$^{\#}$	36.1 ± 0.3$^{\#}$	35.9 ± 0.2	
	V	35.9 ± 0.2	36.4 ± 0.2$^{\#}$	35.9 ± 0.2	36.0 ± 0.3	
	Ph+V	35.8 ± 0.1	36.4 ± 0.5$^{\#}$	36.0 ± 0.3	35.9 ± 0.2	
RBC, x 10^{12} cells/L	Sham	8.0 ± 0.5	7.5 ± 0.4	7.6 ± 0.3	7.7 ± 0.3	0.14
	Ph	7.8 ± 0.4	6.9 ± 1.0$^{\#}$	7.4 ± 0.3	7.8 ± 0.6	
	V	8.9 ± 1.1	7.7 ± 0.3$^{\#}$	7.6 ± 0.8$^{\#}$	7.9 ± 0.4$^{\#}$	

	Ph+V	8.5 ± 1.3	7.3 ± 0.5#	7.4 ± 0.8#	7.9 ± 0.3	
Hgb, g/dL	Sham	13.6 ± 1.1	13.0 ± 0.7	13.4 ± 0.4	13.5 ± 0.66	0.09
	Ph	13.5 ± 0.7	11.8 ± 1.9#	12.9 ± 0.7	13.4 ± 1.2	
	V	13.2 ± 1.0	13.2 ± 0.5	12.8 ± 1.5	13.4 ± 1.0	
	Ph+V	13.5 ± 1.2	12.5 ± 0.6	12.6 ± 1.4	13.7 ± 0.7	
Hematocrit, %	Sham	43.9 ± 2.8	41.0 ± 2.3	42.4 ± 1.8	42.4 ± 2.6	0.70
	Ph	42.3 ± 2.0	37.3 ± 5.5	40.2 ± 2.1	41.4 ± 3.5	
	V	48.4 ± 6.4	42.9 ± 1.8	42.0 ± 4.8	43.4 ± 2.6	
	Ph+V	42.7 ± 14.8	40.3 ± 2.2	40.5 ± 4.4	42.6 ± 2.7	
MCV, fL	Sham	55.0 ± 1.7	55.1 ± 1.8	55.5 ± 2.0	54.7 ± 3.0	0.35
	Ph	54.3 ± 1.8	54.1 ± 1.9	54.1 ± 1.9	53.0 ± 2.2	
	V	54.4 ± 2.1	55.3 ± 1.8	55.3 ± 1.9	54.9 ± 1.8	
	Ph+V	54.7 ± 2.0	55.5 ± 1.5	54.8 ± 1.5	54.2 ± 3.3	
MCHC, g/dL	Sham	30.9 ± 2.2	31.6 ± 1.2	32.1 ± 1.4	32.0 ± 1.4	0.43
	Ph	31.9 ± 1.3	31.7 ± 1.6	32.1 ± 0.8	32.4 ± 1.5	
	V	29.8 ± 1.3	30.8 ± 0.9	30.5 ± 0.7	30.9 ± 1.1	

	Ph+V	31.4 ± 1.7	31.0 ± 0.8	31.1 ± 0.9	32.1 ± 1.4	
Platelets, x 10^9/L	Sham	325 ± 143	535 ± 167$^{\#}$	386 ± 95	479 ± 118	0.23
	Ph	315 ± 94	416 ± 142	427 ± 157	498 ± 139$^{\#}$	
	V	334 ± 202	525 ± 133$^{\#}$	428 ± 133	541 ± 97$^{\#}$	
	Ph+V	431 ± 94	578 ± 147	316 ± 168	588 ± 129	
WBC, x 10^9cells/L	Sham	14.1 ± 4.7	12.3 ± 5.3	10.2 ± 3.5	7.4 ± 2.9	0.08
	Ph	13.9 ± 3.7	13.9 ± 4.1	12.5 ± 4.7	8.2 ± 1.9	
	V	12.1 ± 3.8	12.6 ± 3.7	9.7 ± 2.9	6.7 ± 1.3	
	Ph+V	11.3 ± 3.3	11.1 ± 2.4	13.5 ± 3.9	8.6 ± 3.4	

Lymphocytes, x 10^9 cells/L	Sham	9.5 ± 2.9	6.7 ± 3.0#	7.1 ± 1.9#	5.6 ± 2.1#	0.08
	Ph	9.0 ± 2.3	7.1 ± 3.0	8.2 ± 2.5	5.9 ± 1.4#	
	V	8.2 ± 2.5	7.1 ± 2.0	7.0 ± 1.8	5.0 ± 0.8#	
	Ph+V	7.8 ± 1.6	6.0 ± 1.4	9.0 ± 2.4	5.7 ± 1.6#	
%	Sham	68.7 ± 6.8	53.4 ± 7.7#	70.9 ± 9.7	76.0 ± 3.1	0.10
	Ph	65.7 ± 8.4	49.9 ± 12.3#	67.9 ± 10.1	72.2 ± 6.9	
	V	67.3 ± 5.3	57.6 ± 8.7#	72.8 ± 8.9	75.7 ± 6.1	
	Ph+V	71.1 ± 9.9	55.4 ± 10.9#	67.4 ± 7.4	68.7 ± 10.7	
Monocytes, x 10^9cells/L	Sham	1.0 ± 0.7	0.7 ± 0.7	0.5 ± 0.6	0.2 ± 0.2#	0.25
	Ph	1.1 ± 0.8	1.1 ± 0.8	0.8 ± 0.8	0.2 ± 0.2#	
	V	1.1 ± 0.5	0.6 ± 0.6	0.4 ± 0.6	0.3 ± 0.4#	
	Ph+V	0.7 ± 0.8	0.6 ± 0.6	0.9 ± 0.7	0.4 ± 0.6	
%	Sham	6.6 ± 4.4	5.4 ± 3.8	3.9 ± 4.6	2.2 ± 1.9	0.09
	Ph	7.6 ± 5.3	7.8 ± 4.6	5.9 ± 4.7	2.0 ± 3.3#	
	V	9.1 ± 4.1	5.2 ± 4.2	3.4 ± 4.4#	4.4 ± 5.4	
	Ph+V	5.0 ± 5.1	5.2 ± 4.8	6.0 ± 4.5	3.6 ± 4.9	

Granulocytes,	Sham	3.5 ± 1.4	4.9 ± 2.1	2.7 ± 1.5	1.6 ± 0.7	0.45
x 10^9cells/L	Ph	3.7 ± 1.4	5.5 ± 1.6#	3.5 ± 2.0	2.2 ± 0.8#	
	V	2.9 ± 1.1	4.8 ± 2.3#	2.3 ± 0.9	1.4 ± 0.4#	
	Ph+V	2.8 ± 1.2	4.4 ± 1.8#	3.6 ± 1.4	2.6 ± 1.6	
%	Sham	24.4 ± 4.2	40.1 ± 7.1#	24.7 ± 6.6	21.4 ± 3.0	0.34
	Ph	26.7 ± 5.3	42.3 ± 11#	26.2 ± 7.3	25.8 ± 6.9	
	V	23.6 ± 4.2	37.2 ± 10.0#	23.8 ± 6.1	19.9 ± 3.9	
	Ph+V	23.9 ± 6.0	39.5 ± 12.1#	26.5 ± 4.9	27.7 ± 7.4	
Lactate, mmol/L	Sham	1.2 ± 0.6	1.3 ± 0.3	1.3 ± 0.4	2.0 ± 0.4#	0.79
	Ph	1.0 ± 0.3	1.2 ± 0.4	1.2 ± 0.4	1.9 ± 0.5#	
	V	1.0 ± 0.4	1.4 ± 0.8	1.2 ± 0.4	2.0 ± 0.5#	
	Ph+V	0.9 ± 0.2	1.3 ± 0.7	1.4 ± 0.5	2.2 ± 0.8#	
CRP, μg/ml	Sham	440.1 ± 103.1	763.5 ± 202.0#	507.8 ± 149.9	603.9 ± 158.7#	0.13
	Ph	428.8 ± 78.3	641.6 ± 160.3#	427.7 ± 71.4	421.2 ± 43.4	
	V	483.5 ± 92.2	773.9 ± 160.1#	437.7 ± 82.1	450.5 ± 112.9	
	Ph+V	455.9 ± 144.8	643.5 ± 96.0#	371.3 ± 99.7	483.8 ± 112.7	

RBC, red blood cell count; Hgb, haemoglobin; WBC, white blood cell count; CRP, C-reactive protein. Data shows mean ± SD. Significant difference between treatment groups, *P < 0.05. Within-subject significant differences from baseline, # P < 0.05.
