# Supplementary material for: Evaluation of bacteriophage as an adjunct therapy for treatment of peri-prosthetic joint infection caused by Staphylococcus aureus
Source: PLoS One. 2019 Dec 26;14(12):e0226574. doi: 10.1371/journal.pone.0226574 (PMC6932802; doi:10.1371/journal.pone.0226574)
Supplement: S2 Table — Sensitivity screening of individual phage preparations and the StaPhage cocktail was performed for S. aureus isolates recovered from femur and titanium implants of sham-treated animals and animals treated with phage alone (Ph), vancomycin alone (V) or combination therapy (Ph+V) at day 28 post-surgery (n = 8 animals per group), and compared to that of the original, pre-implantation S. aureus strain. (PDF) [file pone.0226574.s003.pdf]

S2 Table. Phage sensitivity patterns of *S. aureus* isolates. Sensitivity screening of individual phage preparations and the StaPhage cocktail was performed for *S. aureus* isolates recovered from femur and titanium implants of sham-treated animals and animals treated with phage alone (Ph), vancomycin alone (V) or combination therapy (Ph+V) at day 28 post-surgery (n = 8 animals per group), and compared to that of the original, pre-implantation *S. aureus* strain.

Phage Preparation	Experimental Group	Median Sensitivity Score (Min, Max)	P value^
StaPhage cocktail	Pre-implantation	5 (5, 5)	-
	Sham		
	Femur	5 (4, 5)	0.5
	Titanium implant	4.5 (4, 5)	0.125
	Ph		
	Femur	4.5 (4, 5)	0.125
	Titanium implant	4.5 (4, 5)	0.125
	V		
	Femur	5 (4, 5)	0.5
	Titanium implant	4.5 (4, 5)	0.125
	Ph+V		
	Femur	5 (4, 5)	0.25
	Titanium implant	5 (4, 5)	0.25
StaPh_1	Pre-implantation	2 (2, 2)	-
	Sham		
	Femur	2 (1, 2)	0.25
	Titanium implant	2 (1, 2)	0.25
	Ph		
	Femur	1.5 (1, 2)	0.125
	Titanium implant	1.5 (1, 2)	0.125
	V		
	Femur	1 (1, 2)	0.031*
	Titanium implant	1 (1, 2)	0.031*
	Ph+V		
	Femur	1 (1, 2)	0.031*
	Titanium implant	1 (1, 2)	0.016*
StaPh_3	Pre-implantation	5 (5, 5)	-
	Sham		
	Femur	4 (4, 5)	0.031*
	Titanium implant	4 (4, 5)	0.031*
	Ph		
	Femur	5 (4, 5)	0.25
	Titanium implant	4 (4, 5)	0.063
	V		
	Femur	4.5 (4, 5)	0.125
	Titanium implant	4 (4, 5)	0.031*
	Ph+V		

	Femur<br>Titanium implant	4.5 (4, 5)<br>4 (4, 5)	0.125<br>0.063
StaPh_4	Pre-implantation	4 (4, 5)	-
	Sham<br>Femur<br>Titanium implant	 4 (4, 5)<br>4 (4, 5)	 0.5<br>>0.999
	Ph<br>Femur<br>Titanium implant	 5 (4, 5)<br>5 (4, 5)	 0.031*<br>0.063
	V<br>Femur<br>Titanium implant	 4 (4, 5)<br>4.5 (4, 5)	 0.25<br>0.125
	Ph+V<br>Femur<br>Titanium implant	 4 (4, 5)<br>4.5 (4, 5)	 0.25<br>0.125
StaPh_11	Pre-implantation	4 (4, 5)	-
	Sham<br>Femur<br>Titanium implant	 4 (4, 5)<br>4 (4, 5)	 0.5<br>>0.999
	Ph<br>Femur<br>Titanium implant	 5 (4, 5)<br>5 (4, 5)	 0.031*<br>0.063
	V<br>Femur<br>Titanium implant	 4 (4, 5)<br>4.5 (4, 5)	 0.25<br>0.125
	Ph+V<br>Femur<br>Titanium implant	 4 (4, 5)<br>4.5 (4, 5)	 0.25<br>0.125
StaPh_16	Pre-surgery	4 (4, 5)	-
	Sham<br>Femur<br>Titanium implant	 4 (4, 5)<br>4 (4, 5)	 0.5<br>>0.999
	Ph<br>Femur<br>Titanium implant	 5 (4, 5)<br>5 (4, 5)	 0.031*<br>0.063
	V<br>Femur<br>Titanium implant	 4 (4, 5)<br>4.5 (4, 5)	 0.25<br>0.125
	Ph+V<br>Femur<br>Titanium implant	 4 (4, 5)<br>4.5 (4, 5)	 0.25<br>0.125

Statistical analysis performed using Mann-Whitney U test. *P < 0.05 compared to compared to pre-implantation score.
